# Cardiovascular risk in patients visiting the Hadassah AIDS Center, Jerusalem

**DOI:** 10.1186/1758-2652-13-S4-P64

**Published:** 2010-11-08

**Authors:** S Schulman, V Rotshild, K Olshtein Pops, M Korem, S Israel, M Hauzi, S Maayan

**Affiliations:** 1 The Hebrew University of Jerusalem, School of Medicine, Jerusalem, Israel; 2 Hadassah University Hospital, Pharmacy Department, Jerusalem, Israel; 3 Hadassah University Hospital, AIDS Center and Department of Microbiology, Jerusalem, Israel

## Objective

To evaluate cardiovascular risk (CVR) in patients visiting Hadassah AIDS Center & to characterize clinical parameters of patients with high CVR*.*

## Materials and methods

A cross-sectional study. CVR was calculated using Framingham risk score (FRS). Metabolic syndrome (MS) and LDL-cholesterol optimal levels were defined using National Cholesterol Education Program criteria. Adherence to ART was evaluated using the self-reported Visual Analogue Scale.

## Results

*We* analyzed data of 150/350 clinic consecutive patients (median age 41 years, range 24-79; 60% males). Sub-Saharan Africans comprised 51% of patients, mainly immigrants from Ethiopia. Most patients (90%) were on ART, 62% were treated with PIs. Median time for ART and PI exposure were 7 and 4 years, respectively. Most patients (88%) defined adherence to ART>90%. High adherence correlated with viral suppression (p=0.039), but not with increase in CD4. Analyzing traditional CVRs, we observed higher rate of hypertension (HTN) among HIV+ patients compared to the general Israeli population (20% vs. 15%, respectively). In 16% of Ethiopians HTN was likewise observed. HTN rates were 20% among ART-experienced patients, 22% in PI-exposed patients, and 16% among ART-naïve patients. The prevalence of diabetes was 5.7%, similar to the general Israeli population. It was higher among Ethiopians (8%). The rate of smoking was 25% in HIV+ patients, similar to general population (24%) and lower in Ethiopians (8%). Overall increased CVR was observed in 21% of all patients (FRS ≥10%). In 13% the MS was diagnosed. Lower rate of increased CVR (11% only) was observed among HIV+ Ethiopians. Increased CVR was correlated with increased age (p<0.05), male gender (p=0.034), HTN (p=0.002), but not with smoking (p=0.53), change of CD4 (p=0.7) or viral suppression (p=0.64). Increased duration of HIV infection and longer exposure to ART were noted in patients with increased FRS (p=0.059 and p=0.06, respectively), but not so for PI exposure (p=0.1). In 17% of patients LDL-cholesterol levels were higher than optimal goal, as set for CVR factors, especially in the group with increased FRS (p<0.05). Including HIV infection & ART per se as independent CVR, led to LDL goal above target in 30% of the patients in this cohort.

## Conclusions

High rate of HTN and increased CVR were seen in this, mostly ART experienced cohort in Jerusalem. High diabetes prevalence, but lower overall rate of increased CVR were observed among Ethiopian HIV+ patients.

